# Chromosome-Level Genome Assembly of *Cerasus humilis* Using PacBio and Hi-C Technologies

**DOI:** 10.3389/fgene.2020.00956

**Published:** 2020-10-06

**Authors:** Pengfei Wang, Shaokui Yi, Xiaopeng Mu, Jiancheng Zhang, Junjie Du

**Affiliations:** ^1^College of Horticulture, Shanxi Agricultural University, Taigu, China; ^2^College of Life Sciences, Huzhou University, Huzhou, China; ^3^Shanxi Key Laboratory of Germplasm Improvement and Utilization in Pomology, Taigu, China

**Keywords:** *Cerasus humilis*, genome assembly, PacBio, Hi-C, chromosome

## Introduction

**Figure 1:**
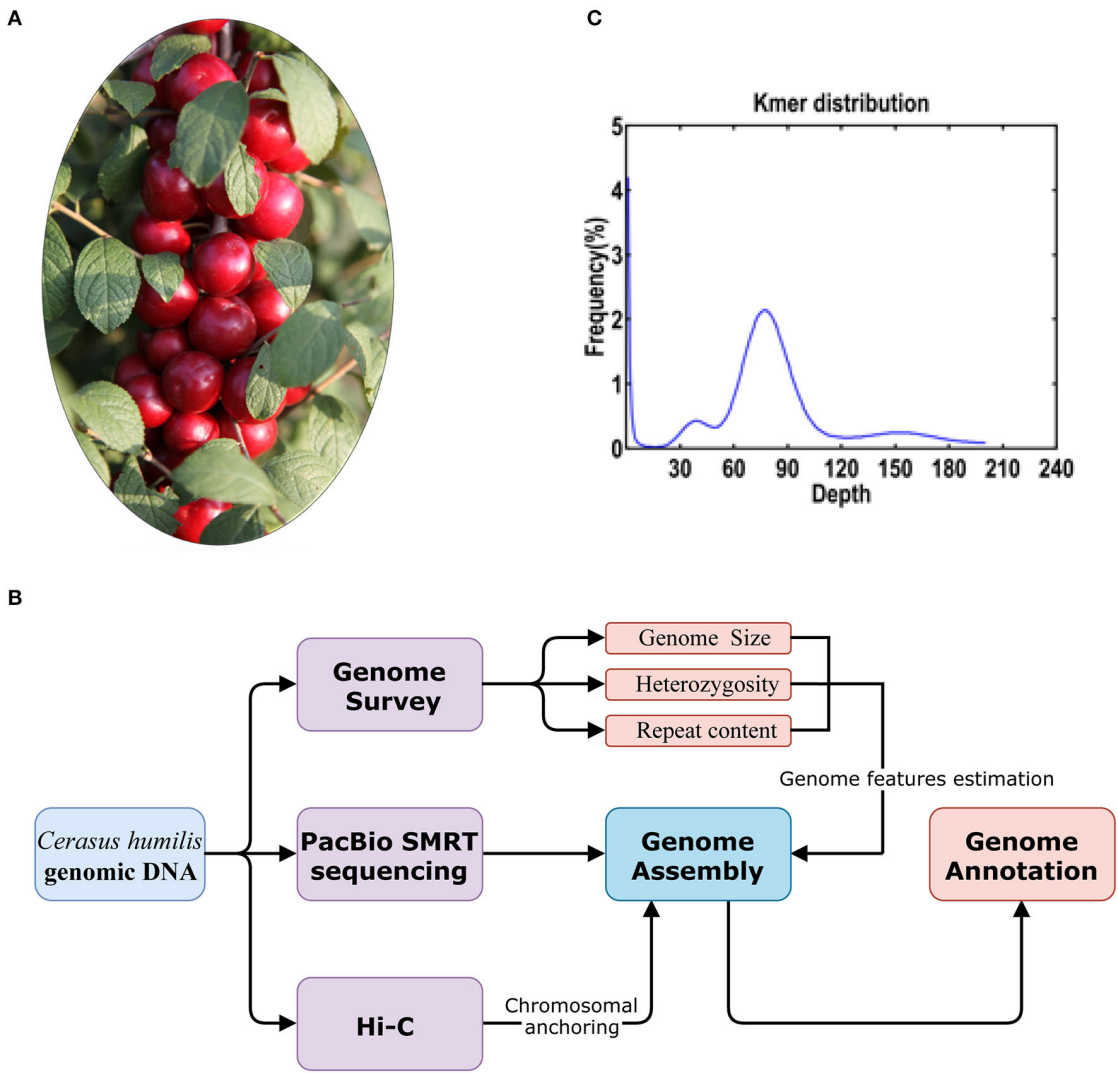
**(A)** The *C. humilis* individual used for whole genome sequencing; **(B)** the workflow of the genome sequencing and analyses and **(C)** distribution of the number of the distinct K-mer (*K* = 17).

The Chinese dwarf cherry (*Cerasus humilis*) is a perennial woody fruit (Figure 1A) native to northern China (Du et al., 1993; Wang et al., 2011; Mu et al., 2016). The fruits of *C. humilis* are red, yellow, green, and purple and have nutritional value (Li et al., 2014; Mo et al., 2015; Wang et al., 2018). The seed kernels of *C. humilis* have been used as a traditional medicine for more than 2000 years in China (Mu et al., 2015). Besides applications in medicine, the Chinese dwarf cherry could potentially have other benefits, including the control of soil erosion, due to its adaptable nature and ability to grow in soils with high salinity and low moisture levels.

Unlike most fruit trees in the genus of *Prunus, C. humilis* typically reaches a height of 0.5–1.2 m and has no obvious juvenile period. Flowering and the production of fruit typically start in the second year after seed sowing. In 2016, an efficient *Agrobacterium*-mediated genetic transformation system was successfully established by Mu et al. (2016) and transgenic *C. humilis* plants showed a significant improvement in rooting abilities. This indicates that *C. humilis* has great potential as a model plant in genetic studies of the genus *Prunus*, particularly since *C. humilis* provides an ideal material for the investigation of genetic regulation of fruit quality due to it has various fruit variations.

Genetic improvement is becoming more and more important as there is an increasing demand for elite cultivars of *C. humilis*. However, due to a lack of genomic information, the current breeding process cannot keep up with the rapid expansion of cultivation. Previously, the chromosome number of this genus has been proved to be 2 × = 16 (Ochatt and Patatochatt, 1994; Verde et al., 2013). The chromosome number of *C. humilis* has been clarified by Wang et al. (2020) in a study that provided a chromosome-level reference genome of *C. humilis* using a combination of the PacBio single molecule real-time (SMRT) sequencing and high-throughput chromosome conformation capture (Hi-C) technologies (Figure 1B).

## Data

A total of 20.86 Gb Illumina short reads from the library were generated to calculate the distribution of *K*-mer depth. Based on the *K*-mer (*K* = 17) distribution, the estimated genome size of *C. humilis* is 228.20 Mb (Figure 1C). Heterozygosity and repeat content were 0.36 and 45.5%, respectively. Subsequently, we assembled the genome sequences into 1,021 contigs with a total length of 229.01 Mb and a contig N50 length of 1.45 Mb with 21.98 Gb PacBio SMRT reads (Table 1). Based on the estimated genome size (~228.20 Mb), the average sequencing coverage was estimated as 96 × . Based on the 35.08 Gb Hi-C clean data, 661 contigs were anchored into 8 pseudo-chromosomes with a total length of 223.46 Mb (97.58% of the total length) (Supplementary Figure 1). The results of quality evaluation for Hi-C data are shown in Supplementary Table 1.

**Table 1:** Summary of obtained sequencing data generated from multiple sequencing technologies for *Cerasus humilis* genome assembly.

**Library type**	**Raw bases (Gb)**	**Insert size (bp)**	**Clean data (Gb)**	**Average read length (bp)**	**Sequencing coverage (X)**
Illumina	21.94	300	20.86	150	91.43
PacBio	21.98	20,000	21.98	8,310	96
Hi-C	35.15	–	35.09	150	153.90

After the misjoin correction and scaffolding with Hi-C data, we obtained a genome with 229.04 Mb in length. The resulting genome assembly was polished using NextPolish software (Hu et al., 2020) with the Illumina short reads used in the genome survey analysis. Finally, a ~229.21 Mb genome with N50 of 26.23 Mb was obtained, which contains 719 contigs. After genome assembly, a total of 26,821 protein-coding genes and 2,233 ncRNAs were identified in the genome. Of these, 16,096, 22,082, and 15,273 predicted genes were functionally annotated using the Swiss-Prot, TrEMBL, and Pfam database, respectively. The average number of exons in the mRNA was 7 and the average length of protein sequences was 331 bp. The repeat elements accounted for 43.1% of the assembly (Supplementary Table 2), which is close to those of the published *Prunus* genomes (Zhang et al., 2012; Jiang et al., 2019).

A total of 100.83 Mb repeat sequences were identified, including 93.36 Mb of interspersed repeats and 7.47 Mb of tandem repeats. Among classified interspersed repeats, retrotransposons (23.70%) were more abundant than DNA transposons (17.10%). Genome completeness was evaluated using the Benchmarking Universal Single-Copy Orthologs (BUSCO) method (Simao et al., 2015). The genes were compared with the BUSCO Embryophyta Odb10 dataset (release 2019-11-20). The results revealed that 98.3% of 1614 conserved orthologous were identified as complete genes. The “complete and single-copy BUSCOs” genes accounted for 94.3% of the total genes, and the “complete and duplicated BUSCOs” genes represented 4.0%.

**Figure 2:**
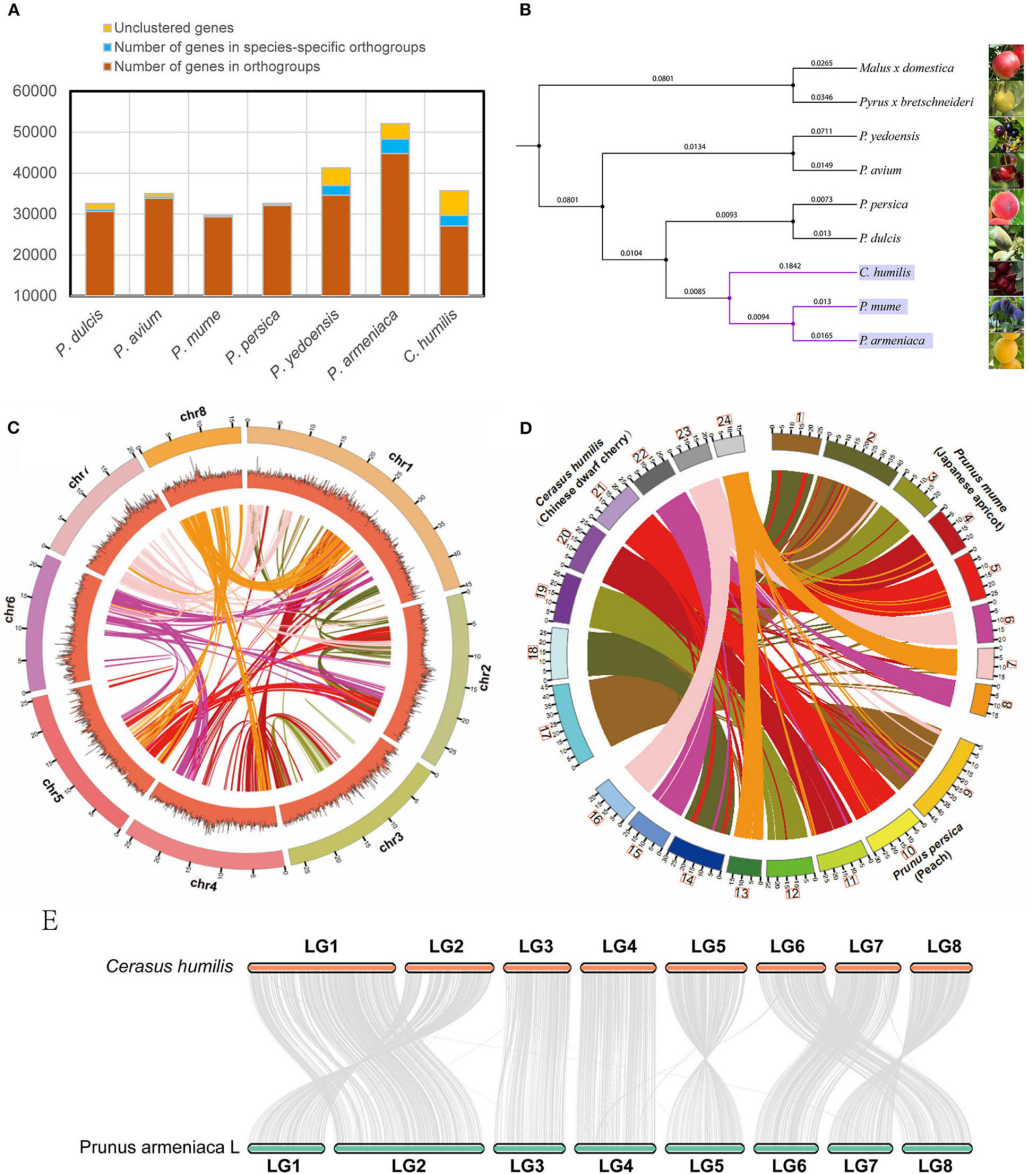
Comparative genomics of *C. humilis* genome. **(A)** the statistics of the gene family for *Prunus* species and **(B)** the phylogenetic tree of 9 closely related species. **(C)** Synteny distribution of the 8 pseudo-chromosomes of *C. humilis*; The tracks indicate chromosomes (Chr) and GC contents, respectively. **(D)** Comparative synteny analysis between *C. humilis, P. mume*, and *P. persica*; Putative homologous synteny blocks among three genomes are linked with lines, which were colored according to *C. humilis* genome chromosomes. **(E)** Syntenic comparison of the homologous chromosomes between *C. humilis* and *P. armeniaca*.

We aligned the *C. humilis* genome with the available genomes of 8 close-related species. In *C. humilis*, 26,821 genes were clustered into 19,200 gene families (Figure 2A). Gene family analysis also revealed that 973 gene families and 2,580 genes were unique to *C. humilis* in the comparison. On the other hand, the *Prunus armeniaca, P. yedoensis*, and *C. humilis* presented more species-specific gene families (Supplementary Table 3). A phylogenetic tree was constructed based on single-copy orthologous (Figure 2B) and the result indicated that *C. humilis* was more closely related to *P. armeniaca* (apricot) and *P. mume* (Japanese apricot), and this clade showed a closer relationship with *P. persica* and *P. dulcisi*, which coincides with results observed in a previous study by Jiang et al. (2019).

To further evaluate the quality of this genome assembly, we compared *C. humilis* with the genomes of *P. persica* (Peach), *P. mume* (Japanese apricot), and *P. armeniaca* (Apricot), which are the closest species with a chromosome-level assembly. Firstly, the conservation synteny among the 8 pseudo-chromosomes of *C. humilis* was investigated. A total of 2,195 homologous synteny blocks were detected between pseudo-chromosomes, and some homologous blocks within pseudo-chromosomes were also observed (Figure 2C). Meanwhile, the gene synteny among the genomes of *C. humilis, P. mume*, and *P. persica* were compared and a highly conserved synteny and strict correspondence of chromosome assignment were observed among these three species (Figure 2D). The chromosome synteny of *C. humilis* and apricot showed that these species exhibited high collinearity and a relatively low frequency of fragment rearrangements was observed between these two species (Figure 2E).

## Materials and Methods

### Sample Collection

The leaves of a 10-year-old *C. humilis* individual were collected in 2017 at the Shanxi Germplasm Bank of *C. humilis* (Taigu, Shanxi, China). Total genomic DNA was extracted using a DNA Extraction Kit (TaKaRa, Dalian, China) following the manufacturer's protocols. The quality and quantity of total DNA were determined with 1% agarose gel electrophoresis and a NanoDrop 2000 spectrophotometer (Thermo Fisher Scientific, MA, USA).

### Genome Feature Estimation Using the K-mer Method

The qualified DNA was randomly disrupted into 350 bp fragments and subjected to construct in the Illumina library using the standard protocol provided by Illumina (San Diego, CA, USA). The paired-end sequencing was performed using the Illumina HiSeq 4000 system with paired-end 150 bp (PE150). After the quality control of raw data, a total of 20.86 Gb clean reads were generated and used for the estimation of genome size. We calculated the number of 17-mer from the clean reads using the Jellyfish version 2.0 (Marcais and Kingsford, 2011). The genome size and heterozygosity were evaluated based on the peaks of 17-mer distribution.

### Libraries Construction and PacBio, Hi-C Sequencing

Genomic DNA was used for the library construction for sequencing on the PacBio Sequel System. Three 10 Kb libraries were constructed following the PacBio manufacturing protocols, and then the libraries were sequenced with two cells on the PacBio Sequel System. Meanwhile, the Hi-C technique was used for constructing the chromosome-level assembly of *C. humilis*. The sample was fixed with fresh formaldehyde and then DNA-protein bonds were created. The restriction enzyme of *Mbo* I was selected to digest the DNA and the overhanging 5′ ends of the DNA fragments were repaired with a biotinylated residue. The fragments that were close to each other in the nucleus during fixation were then ligated. The Hi-C fragments were further sheared by sonication into smaller fragments of ~350 bp in size, which were then pulled-down with streptavidin beads. The Hi-C library for Illumina sequencing was prepared according to the manufacturer's standard procedures. The library was sequenced on the Illumina HiSeq 4000 platform with PE150.

### Genome Assembly Based on PacBio and Hi-C Data

Of the raw reads generated from the PacBio platform, we removed those containing adaptor sequences or low-quality reads. The remaining reads were processed by self-correction using Falcon v1.8.2 (Chin et al., 2016). We processed the genome assembly based on these error-corrected reads, detecting overlaps among reads, and assembling the final string graph following the Falcon pipeline. To obtain chromosome-level scaffolds, Hi-C raw reads generated from the Illumina platform were filtered and then used for subsequent analyses. They were mapped to the assembled contigs for constructing the contacts among the contigs using BWA v0.7.10 (Li and Durbin, 2009) with default parameters. The HiC-Pro software (Servant et al., 2015) was used to identify the valid interaction pairs of the unique mapping reads and the invalid interaction pairs. Subsequently, LACHESIS v2.27 software (Burton et al., 2013) was used for the ultra-long-range scaffolding of *de novo* genome assemblies using the signal of genomic proximity provided by the Hi-C data. After the assembly, NextPolish was used to polish the assembled contigs with the Illumina short reads used in genome survey analysis.

### Repeats and Gene Annotation

We masked the repetitive regions of the assembled genome sequences using the REPET program (Flutre et al., 2011). For protein-coding gene prediction, we used both homology-based and *de novo* strategies following the Maker pipeline (Cantarel et al., 2007) to predict genes in the genome. The *ab intio* gene prediction was performed on the repeat-masked genome assembly using SNAP (Korf, 2004) and Augustus (Stanke et al., 2008). For homology-based prediction, we mapped the protein sequences of *Prunus avium, P. persica, Arabidopsis thaliana*, and *Glycine max* onto the generated assembly using BLASTX with an *E*-value of 10^−5^. The homology alignment of protein-coding genes was performed with public protein databases, including Swiss-Prot, TrEMBL, and Pfam.

### Gene Family and Phylogenetic Tree Construction

The protein sequences of *C. humilis* and eight closely related species (*P. armeniaca* L., *P. yedoenis, P. persica (L), P. avium* (L) L., *P. mume* (mei), *P. dulcis* Miller., *Malus domestica* Borkh., and *Pyrus bretschneideri* Rehder) were used to analyze gene families. An all-to-all BLASTP analysis of proteins with a length ≥50 amino acid (aa) was performed with an *E*-value of 10^−5^. The paralogous and orthologous genes were identified using OrthoFinder software (Emms and Kelly, 2015). The single-copy orthologs were used to construct the phylogenetic tree. The species tree inference was performed with the STAG algorithm based on the concatenated multiple sequence alignment.

### Chromosome Evolution and Collinear Analysis

To investigate the chromosome evolution between *C. humilis* and its closely related species, collinearity analyses were performed using the MCScan toolkit implemented in Python (https://github.com/tanghaibao/jcvi). The conservation synteny among the eight pseudo-chromosomes of *C. humilis* was investigated. Meanwhile, the gene synteny among the genomes of *C. humilis, P. mume*, and *P. persica* were compared. Given that apricot is the closest species of *C. humilis* based on the result of the species tree, we compared the chromosome synteny of *C. humilis* and apricot.

## Data Availability Statement

The raw reads generated from Illumina platform, PacBio long reads and Hi-C data have been deposited in NCBI Sequence Read Archive (SRA) under the accession number SRR10912179, SRR10913200 and SRR10882935, respectively. The Illumina RNA173 seq data used for genome annotation was deposited in the NCBI SRA under the accession number: SRR10913940 SRR10913942. The sequences of genome assembly are available in the figshare with https://doi.org/10.6084/m9.figshare.11669673. The GFF file is deposit at the figshare with https://doi.org/10.6084/m9.figshare.11669514.

## Author Contributions

PW, XM, and JD conceived the study. SY performed bioinformatics analysis. PW and XM collected the samples and extracted the genomic DNA. XM, SY, and PW wrote the manuscript. JZ revised the manuscript. All authors read and approved the final manuscript. All authors contributed to the article and approved the submitted version.

## Conflict of Interest

The authors declare that the research was conducted in the absence of any commercial or financial relationships that could be construed as a potential conflict of interest.

## Supplementary Material

Supplementary Figure 1 The contact matrix of the *C. humilis* genome contigs using Hi-C data. The color bar indicates the logarithm of the contact density from red (high) to white (low) in the plot.

Supplementary Table 1 The quality evaluation of Hi-C data used for genome assembly.

Supplementary Table 2 The repeat elements identified in the *C. humilis* genome.

Supplementary Table 3 The statistics of the gene family in 9 closely related species.
