# Supplementary figures and images for: Chromosome-Level Genome Assembly of Cerasus humilis Using PacBio and Hi-C Technologies
Source: Front Genet. 2020 Oct 6;11:956. doi: 10.3389/fgene.2020.00956 (PMC7573120; doi:10.3389/fgene.2020.00956)

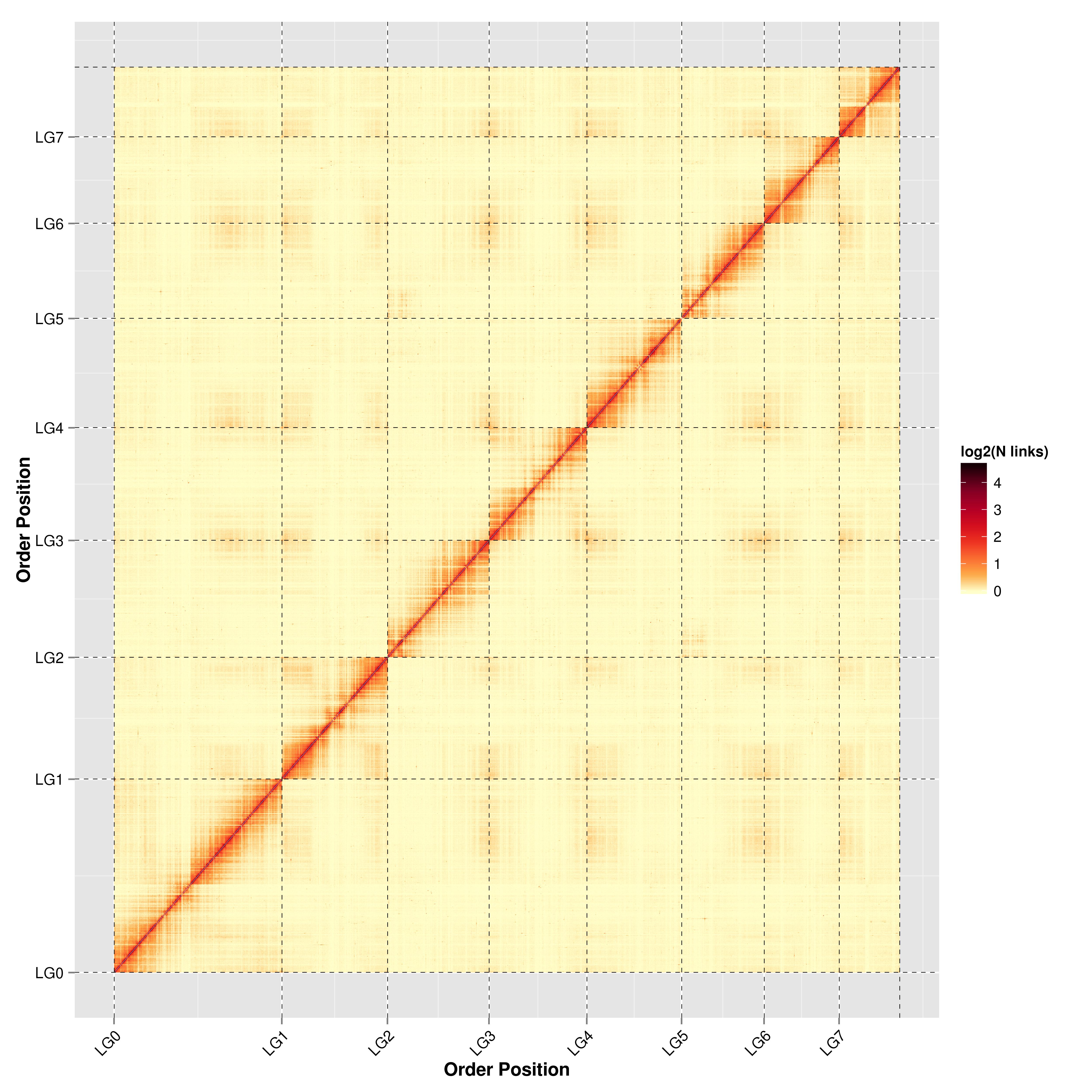

Supplement: Supplementary Figure 1 — The contact matrix of the C. humilis genome contigs using Hi-C data. The color bar indicates the logarithm of the contact density from red (high) to white (low) in the plot. [file Image_1.JPEG]
