# Supplementary material for: Chromosome-Level Genome Assembly of Cerasus humilis Using PacBio and Hi-C Technologies
Source: Front Genet. 2020 Oct 6;11:956. doi: 10.3389/fgene.2020.00956 (PMC7573120; doi:10.3389/fgene.2020.00956)
Supplement: Supplementary Table 1 — The quality evaluation of Hi-C data used for genome assembly. [file Table_1.DOCX]

| Type | Number | Ratio （%） |
| --- | --- | --- |
| Total Read Pairs | 117,163,983 | 100 |
| Mapped Reads | 200,333,620 | 85.49 |
| Unique Mapped Read Pairs | 62,329,353 | 53.2 |
| Unique Paired Alignments | 62,329,353 | 100 |
| Valid Interaction Pairs | 51,931,633 | 83.32 |
| DangLing End Pairs | 2,495,330 | 4 |
| Re-ligation Pairs | 1,035,069 | 1.66 |
| Self-cycle pairs | 3,698,991 | 5.93 |
| Dumped Pairs | 3,168,330 | 5.08 |

Table S1 The quality evaluation of Hi-C data used for genome aseembly.
