# Supplementary material for: Chromosome-Level Genome Assembly of Cerasus humilis Using PacBio and Hi-C Technologies
Source: Front Genet. 2020 Oct 6;11:956. doi: 10.3389/fgene.2020.00956 (PMC7573120; doi:10.3389/fgene.2020.00956)
Supplement: Supplementary Table 2 — The repeat elements identified in the C. humilis genome. [file Table_2.DOCX]

**Table** **S2** Repeat content in *Cerasus humilis* genome

| **Class** | **Order** | **Wicker's Code** | **Base pairs** | **Percentage of genome** |
| --- | --- | --- | --- | --- |
| **Retrotransposon** | LINE | RIX-incomp | 3,068,099 | 1.30% |
|  |  | RIX-incomp-chim | 4,068 | 0 |
|  | LTR | RLX-incomp | 7,079,966 | 3.10% |
|  |  | RLX-incomp-chim | 291,912 | 0.10% |
|  | SINE | RSX-incomp | 359,597 | 0.20% |
|  |  | RSX-incomp-chim | 232712 | 0.10% |
|  | - | RXX-LARD | 11,641,638 | 5.10% |
|  |  | RXX-LARD-chim | 5,936 | 0 |
|  | - | RXX-TRIM | 1,332,427 | 0.60% |
|  |  | RXX-TRIM-chim | 43,193 | 0 |
|  | - | RXX | 18,179,933 | 8% |
|  |  | RXX-chim | 83,333 | 0 |
|  | DIRS | RYX-incomp | 9,259,741 | 4% |
|  |  | RYX-incomp-chim | 2,731,321 | 1.20% |
| **DNA transposon** | TIR | DTX-incomp | 19,464,677 | 8.50% |
|  |  | DTX-incomp-chim | 1,312,782 | 0.60% |
|  | - | DXX-MITE | 2,344,137 | 1% |
|  | Helitron | DHX-incomp | 387,515 | 0.20% |
|  | Maverick | DMX-incomp | 1,147,329 | 0.50% |
|  |  | DMX-incomp-chim | 4,315,181 | 1.90% |
|  | - | DXX-chim | 1,543,206 | 0.70% |
|  | - | XXX-chim | 250,335 | 0.10% |
|  | - | noCat | 8,316,914 | 3.60% |
| **TE** | - | - | 93,359,952 | 40.80% |
| **SSR** | - | - | 7,474,623 | 3.30% |
